# *Allomyrina Dichotoma* Larvae Regulate Food Intake and Body Weight in High Fat Diet-Induced Obese Mice Through mTOR and Mapk Signaling Pathways

**DOI:** 10.3390/nu8020100

**Published:** 2016-02-18

**Authors:** Jongwan Kim, Eun-Young Yun, Seong-Won Park, Tae-Won Goo, Minchul Seo

**Affiliations:** 1 Department of Anatomy, Graduate School of Dongguk University College of Medicine, Gyeongju 38066, Korea; kimjw3189@naver.com; 2 Department of Agricultural Biology, National Academy of Agricultural Science, RDA, Wanju-gun 55365, Korea; yuney@korea.kr; 3 Department of Biotechnology, Catholic University of Daegu, Daegu 38430, Korea; microsw@cu.ac.kr; 4 Department of Biochemistry, Dongguk University College of Medicine, Gyeongju 38066, Korea

**Keywords:** *Allomyrina dichotoma* larvae, diet-induced obesity, ER stress, hypothalamus, appetite

## Abstract

Recent evidence has suggested that *the Korean horn beetle (Allomyrina dichotoma)* has anti-hepatofibrotic, anti-neoplastic, and antibiotic effects and *is* recognized as a traditional medicine. In our previous works, *Allomyrina dichotoma* larvae (ADL) inhibited differentiation of adipocytes both *in vitro* and *in vivo*. However, the anorexigenic and endoplasmic reticulum(ER) stress-reducing effects of ADL in obesity has not been examined. In this study, we investigated the anorexigenic and ER stress-reducing effects of ADL in the hypothalamus of diet-induced obese (DIO) mice. Intracerebroventricular (ICV) administration of ethanol extract of ADL (ADE) suggested that an antagonizing effect on ghrelin-induced feeding behavior through the mTOR and MAPK signaling pathways. Especially, ADE resulted in strong reduction of ER stress both *in vitro* and *in vivo*. These findings strongly suggest that ADE and its constituent bioactive compounds are available and valuable to use for treatment of various diseases driven by prolonged ER stress.

## 1. Introduction

Obesity is widely recognized as the largest and fastest growing public health problem in the world. Obesity is caused by an imbalance between energy intake and expenditure and results in major comorbidities, including metabolic syndrome, hypertension, type 2 diabetes, stroke, cancer, and dyslipidemia [1,2,3]. Although anti-obesity drugs were heralded as an answer to the obesity problem in the past, they have demonstrated inconsistency and side effects. Therefore, new treatments for obesity that are both better tolerated and more efficacious are urgently needed. For decades, bioactive products have been identified and isolated from a variety of sources such as living organisms and are widely used in traditional medicine and the food industry [4,5]. Among them, insects have gained attention as a source of effective bioactive products [6]. Although there is a lack of scientific evidence regarding the safety and beneficial effects of insects, numerous insect species are used as a traditional food or medicine in many countries.

*Allomyrinal dichotoma* (*A. dichotoma*) is a species of rhinoceros beetle and widely used in traditional medicine for its anti-diabetic, anti-hepatofibrotic, anti-neoplastic, and anti-obesity effects [4,5,7,8]. The Food and Agriculture Organization of the United Nations (FAO) reported the possibility of using edible insects in human dietary supplements in the future. Despite growing interest in insect-based bioactive products, the biological activities of insect-based products have rarely been studied. In previous studies, we reported that *A. dichotoma* larvae (ADL) inhibit *in vitro* and *in vivo* differentiation of adipocytes via downregulation of transcription factors, peroxisome proliferator-activated receptor-γ (PPARγ), and CCAAT/enhancer binding protein-α (C/EBPα) [7,9].

The hypothalamus plays a central role in the regulation of feeding behavior and energy balance by controlling appetite regulatory neuropeptides and specific neuronal excitations [10,11]. However, disruption of these physiological functions of the hypothalamus has been implicated in various diseases, such as diabetes mellitus, neurodegenerative disease, ischemia, prion disease, and cystic fibrosis [12,13]. In particular, hypothalamic ER stress has been suggested to cause feeding behavior disorder and glucose dysregulation for promotion of obesity and diabetes [14,15,16]. Therefore, sustaining the functional roles of hypothalamic neurons may be beneficial for hypothalamic regulation of energy balance.

In the present study, we investigated the appetite regulatory effect of natural products extracted from *A. dichotoma* larvae (ADE) on the hypothalamus, a section of the brain responsible for homeostasis, of high-fat-induced obese mice since there has been no study elucidating the direct effect of ADE on appetite control in the hypothalamus. We determined food intake, body weight, appetite regulatory neuropeptides, and ER stress in mice fed high-fat diets with or without ADE. Our results demonstrate the potential of ADE as a novel treatment option for anorexigenic function in high-fat-induced obese mice via reduction of ER stress.

## 2. Materials and Methods

### 2.1. Reagents and Cells

DMSO was purchased from Sigma-Aldrich (Sigma-Aldrich, St Louis, MO, USA). QGreenTM 2X SybrGreen qPCR Master Mix was purchased from CellSafe (CellSafe, Suwon, Korea). Mouse hypothalamic GT1-7 cells were maintained in Dulbecco’s modified Eagle’s media (Gibco, Rockville, MD, USA) supplemented with 10% heat-inactivated fetal bovine serum (FBS) (Gibco, Grand Island, NY, USA), 100 U/mL of penicillin, and 100 μg/mL of streptomycin (Gibco, Grand Island, NY, USA).

### 2.2. Preparation of Allomyrina Dichotoma Larvae Extract (ADE)

Freeze-dried ADL (fdADL), ground to a powder and sterilized, was provided by World Way Co. (Yeongi, Korea). fdADL was mixed with ethanol (1 g of fdADL/mL of ethanol) and incubated at RT for 30 min after ultrasonication (250 J, 10 s, two times). After incubation, the supernatant was filtered and completely dried using a rotary evaporator. Dried ethanol extract of ADL was dissolved in 20% DMSO.

### 2.3. MTT Assay

Cell viability was determined by 3-(4,5-dimet hylthiazol-2-yl)-2,5-diphenyltetrazolium bromide (MTT) assay. Hypothalamic neuronal GT1-7 cells were seeded in triplicate at a density of 1 × 10^4^ cells per well on a 96-well plate. After treatment, culture media were removed and MTT (0.5 mg/mL) added, followed by incubation at 37 °C for 2 h in a CO_2_ incubator. After dissolving the insoluble crystals that formed in DMSO, absorbance was measured at 570 nm using a microplate reader (Anthos Labtec Instruments, Wals, Austria).

### 2.4. Mice

Male C57BL/6J mice (7 weeks of age) were obtained from Japan SLC (Hamamatsu, Japan). Mice were allowed free access to standard chow diet and water for 1 week. To generate diet-induced obesity (DIO), 8-week-old mice were fed a high-fat diet (HFD, 60% fat, D12492; Research Diets, New Brunswick, NJ, USA) for 8 weeks. Lean control mice were fed a low-fat diet (LFD, 10% fat, D12450B; Research Diets) for the same period. The mice were placed in a controlled temperature room (23 °C) with a 12-h light/12-h dark cycle with free access to food and water. All procedures followed the Principles of Laboratory Animal Care (NIH, Washington, DC, USA) and were approved by the Institution Animal Care and Use Committee of College of Medicine, Dongguk University.

### 2.5. Intracerebro-Ventricular Cannulation and ADE Administration

Mice were implanted with a 26-gauge stainless guide cannular (5 mm bellow pedestal; C315G, Plastics one, Roanoke, VA, USA) into the third ventricle under stereotaxic control using a stereotaxic apparatus (coordinates from Bregma: anteroventral, −1.8 mm; lateral, 0.0 mm; dorsoventral, 5.0 mm) through a hole created in the skull with a micro driller. The cannula was secured to the skull with dental cement and capped with a dummy cannular (C315DC, Plastic one) that extended 0.5 mm below the guide cannular. Animals were weighed daily, and any animal showing signs of illness or weight loss was removed from the study and euthanized. At 7 days after ICV cannulation, HFD (*n* = 20) and LFD (*n* = 20) mice were divided into two groups. The first group of HFD (*n* = 10) and LFD mice (*n* = 10) was infused with 1 μL of 20% DMSO as a vehicle, whereas the second group of HFD (*n* = 10) and LFD (*n* = 10) mice was infused with 1 μL of ADE (10 mg/mL). All ICV injections were made using a 33-gauge internal cannular (C315I, Plastic one) that extended 0.5 mm below the guide cannular, connected by a cannular connector to a 5 μL Hamilton syringe and infused over 5 min. At 12 h after ADE infusion, hypothalamus were dissected and flash frozen in liquid nitrogen and kept in −80 °C until further processing.

### 2.6. Western Blot Analysis

Tissue or cells were lysed in RIPA lysis buffer (50 mM Tris-HCl, pH 8.0, 150 mM NaCl, 0.02% sodium azide, 0.1% SDS, 1% NP-40, 0.5% sodium deoxycholate, and 1 mM phenylmethylsulfonyl fluoride). Protein concentrations of cell lysates were measured using a Bio-Rad protein assay kit (Bio-Rad, Hercules, CA, USA). Equal amounts of protein were separated by 8% or 12% SDS-PAGE and transferred to PVDF membranes (Bio-rad, Hercules, CA, USA). Membranes were blocked with 5% skim milk and sequentially incubated with primary antibodies (mouse monoclonal anti-CHOP antibody (1:1000; Cell Signaling Technology, Danvers, MA, USA); rabbit polyclonal anti-phospho-eIF2α antibody (1:1000; Cell Signaling Technology, Danvers, MA, USA); rabbit polyclonal anti-eIF2α antibody (1:1000; Cell Signaling Technology, Danvers, MA, USA); mouse monoclonal anti-Ero1L antibody (1:1000; Abnova, Taipei, Taiwan); mouse monoclonal anti-PDI antibody (1:1000; Enzo Life Sciences, Inc., Plymouth Meeting, PA, USA); rabbit monoclonal anti-phospho-Stat3 (Tyr705) (1:1000; Cell Signaling Technology, Danvers, MA, USA); mouse monoclonal anti-Stat3 (1:1000; Cell Signaling Technology, Danvers, MA, USA); rabbit polyclonal anti-SOCS3 antibody (1:1000; Cell Signaling Technology, Danvers, MA, USA); α-tubulin antibody (1:2000; Sigma-Aldrich, St Louis, MO, USA), and HRP-conjugated secondary antibody (1:10,000; anti-mouse and rabbit-IgG antibody; Amersham Biosceinces, Piscataway, NJ, USA )), followed by detection using an ECL detection kit (Invitrogen, Waltham, MA, USA).

### 2.7. Reverse Transcription-PCR

Total RNA was extracted from tissue or cells with an RNeasy Mini Kit (Qiagen, Hilden, Germany), according to the manufacturer’s instructions. An aliquot of RNA was subjected to 1% agarose gel electrophoresis to confirm integrity. cDNA was synthetized with M-MLV Reverse Transcriptase (Promega, Madison, WI, USA) and oligo(dT) primers after DNase I treatment (Invitrogen, Life Technologies). Real-time PCR was performed using the specific primer set in Table 1. Traditional PCR amplification was carried out at an annealing temperature of 60 °C for 27 cycles using the specific primer set in Table 1. For analysis of PCR products, 10 μL of each PCR product was electrophoresed on a 1%–2.5% agarose gel and detected under UV light. Gapdh was used as an internal control.

### 2.8. Data Analysis

All data are presented as the means ± SDs. Comparisons between two groups were performed using the Student’s *t*-test. Comparisons between three or more groups were analyzed using one-way ANOVA with Dunnett experiments. SPSS version 18.0 K (SPSS Inc., Chicago, IL, USA) was used for the analysis, and *p* value differences of < 0.05 were considered statistically significant.

## 3. Results

### 3.1. High Fat Diet-Induced Obesity Induces Hypothalamic Endoplasmic Reticulum Stress

**Figure 1:**
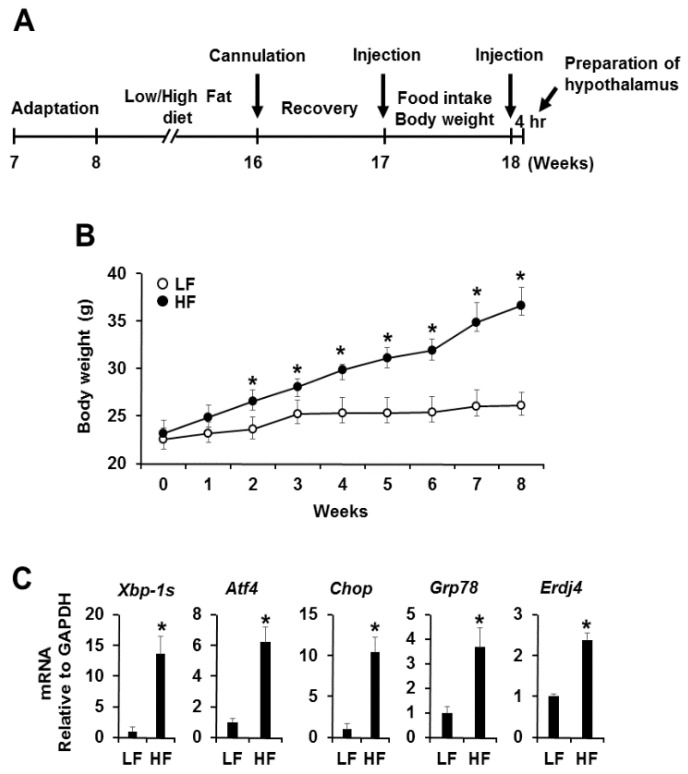
Increased hypothalamic ER stress in mice fed a high-fat diet. (**A**) Experimental timeline of the experimental procedure; (**B**) Time dependence of body weight in low fat and high-fat-diet-induced mice. At 8 weeks of age, mice fed a high-fat diet showed a 43% increase in body weight compared with those fed a low-fat diet. The results are means ± SDs (*n* = 10); * *p* values of < 0.05 indicate significant difference from low-fat diet-induced mice; (**C**) mRNA expression levels of ER stress responsive markers in low fat and high-fat-diet-induced mice. mRNA expression levels of ER stress responsive markers were dramatically upregulated in high-fat-diet-induced obese mice. The results are means ± SDs (*n* = 10); * *p* values of < 0.05 indicate significant difference from low-fat-diet-induced mice. LF, low-fat diet. HF, high-fat diet.

To examine whether obesity induces ER stress in hypothalamus, we fed male C57BL/6J mice a high-fat diet (HFD; 60% kcal from fat) and low-fat diet (LFD; 10% kcal from fat) for 8 weeks (Figure 1A). Body weight significantly increased in high-fat-diet-induced mice during the study period compared with low-fat-diet-induced mice (Figure 1B). We then performed quantitative PCR analysis of ER stress responsive markers, such as spliced X-box binding protein 1 (*Xbp-1s*), activating transcription factor 4 (*Atf4*), c/EBP-homologous protein (*Chop*), 78 kDa glucose-regulated protein (*Grp78*), and ER *DnaJ* homolog 4 (*Erdj4*), after mRNA isolation from the hypothalamus of mice fed a high-fat diet or low-fat diet for 8 weeks. The expression levels of ER stress responsive markers were dramatically upregulated in high-fat-diet-induced mice (Figure 1C). Taken together, these results indicate that the persistence of obesity gradually induces ER stress, followed by activation of the UPR signaling pathway in the hypothalamus.

### 3.2. Central Administration of ADE Reduces Food Intake and Body Weight through Regulation of Appetite-Related Neuropeptides in High Fat Diet-Induced Mice

**Figure 2:**
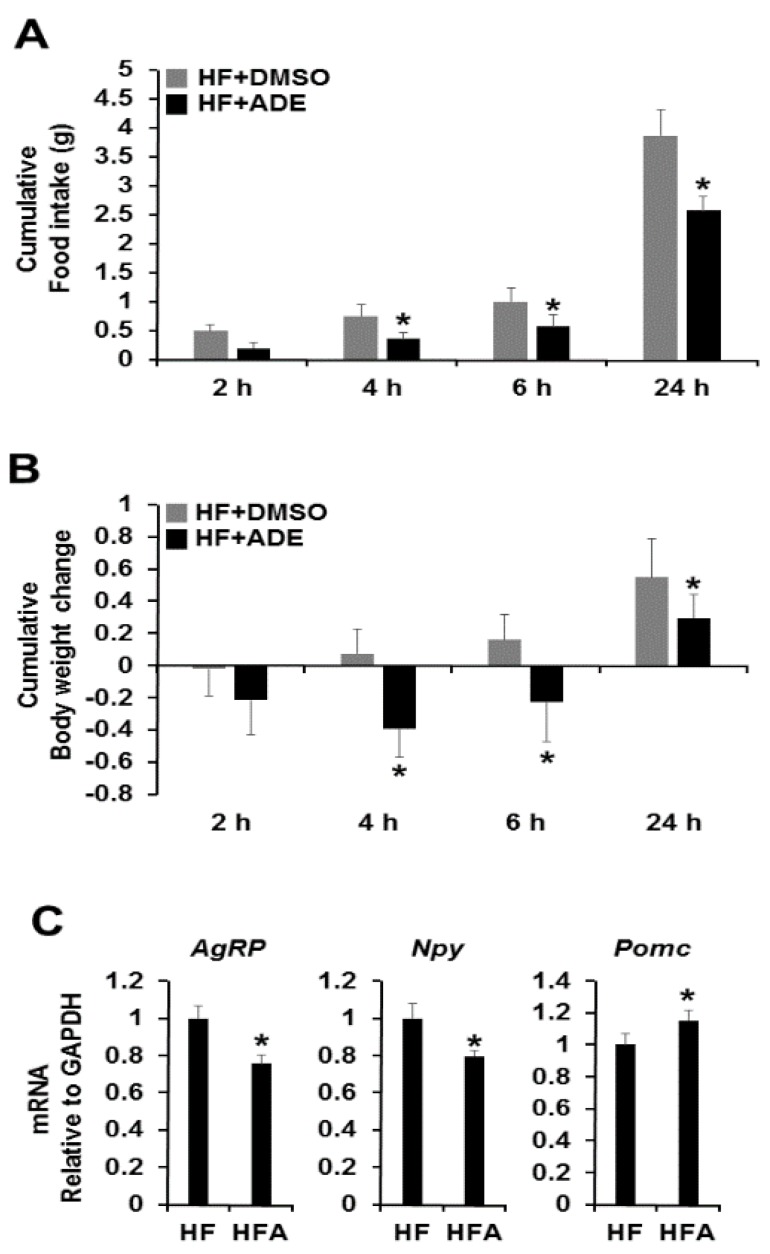
Effects of central administration of ADE on food intake and body weight. The average cumulative food intake (**A**) and body weight (**B**) were measured in high-fat-diet-induced mice ICV administration with ADE (1 μL of 10 mg/mL) or DMSO (1 μL of 20% DMSO) during the experimental period. The results are means ± SDs (*n* = 10 per group); * *p* values of < 0.05 indicate significant difference from administration with DMSO (1 μL of 20% DMSO). (**C**) Effects of ICV administration of ADE (1 μL of 10 mg/mL) on hypothalamic mRNA expression levels of neuropeptides. The results are means ± SDs (*n* = 10 per group); * *p* values of < 0.05 indicate significant difference from administration with DMSO (1 μL of 20% DMSO). ADE, ethanol extract of *Allomyrina dichotoma* larvae. HF, high-fat diet with DMSO. HFA, high-fat diet with ADE.

To examine the possibility that ADE as a natural food supplement can regulate appetite, we tested cytotoxicity of ADE before central administration using hypothalamic neuronal GT1-7 cells. The cytotoxicity of ADE was determined by 3-(4,5-dimethylthiazol-2-yl)-2,5-diphenyltetrazolium bromide (MTT) assay after treatment with various concentrations of ADE (0.01–5 mg/mL). As a result, up to 5 mg/mL of ADE showed no cytotoxic effect on hypothalamic neuronal GT1-7 cells [17]. To determine whether or not central administration of ADE regulates food intake and body weight in high-fat-diet-induced mice, we administrated a single dose of ADE (1 μL of 10 mg/mL ADE) into the third ventricle. Administration of ADE significantly reduced food intake and body weight during 24 h compared to the vehicle control, which was evident 2 h after infusion and was consistent after 24 h (Figure 2A,B, Supplementary Figure S1A,B). Next, we evaluated whether or not the anorexigenic action of ADE is associated with changes in ARC-derived neuropeptides. As shown in Figure 2C, central administration of ADE decreased the mRNA expression levels of *Agrp* and *Npy*, whereas *Pomc* expression increased in high-fat-diet-induced mice. However, low-fat-diet-induced mice showed no changes (Supplementary Figure S1C).

### 3.3. Central Administration of ADE Reduces Hypothalamic Endoplasmic Reticulum Stress

**Figure 3:**
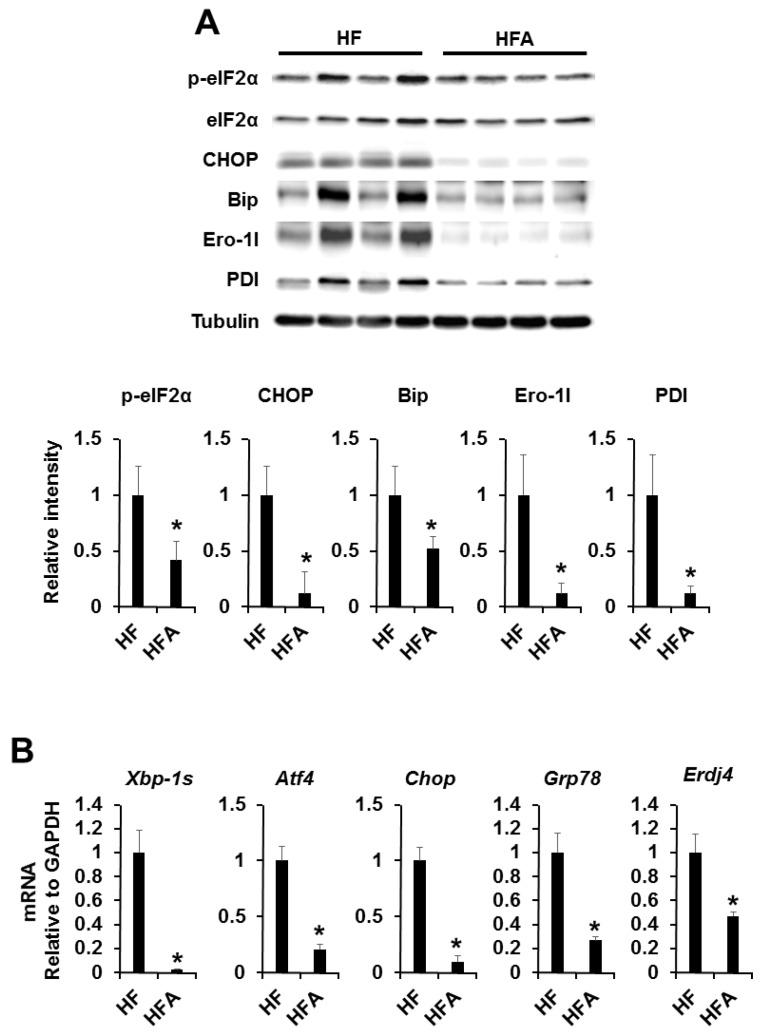
Effects of central administration of ADE on ER stress responsive markers and ER chaperone/foldases expression. (**A**) Effects of ICV administration of ADE (1 μL of 10 mg/mL) on hypothalamic ER stress responsive markers and ER chaperone/foldases. The results of densitometric analysis (*lower*) are means ± SDs (*n* = 10); * *p* values of < 0.05 indicate significant difference from administration with DMSO (1 μL of 20% DMSO); (**B**) Effects of ICV administration of ADE (1 μL of 10 mg/mL) on hypothalamic mRNA expression levels of hypothalamic ER stress responsive markers. The results are means ± SDs (*n* = 10 per group); * *p* values of < 0.05 indicate significant difference from administration with DMSO (1 μL of 20% DMSO). HF, high-fat diet with DMSO. HFA, high-fat diet with ADE.

In previous works, under diet-induced obesity (DIO) conditions, orexigenic neuropeptides (AgRP and NPY) were induced while anorexigenic neuropeptides (α-MSH) were reduced by hypothalamic ER stress [18,19,20]. To examine whether or not ADE can regulate hypothalamic ER stress in high-fat-diet-induced mice, we administrated ADE and vehicle control into the third ventricle of high fat or low-fat-diet-induced obese mice. Expression levels of ER stress responsive markers (phosphor-eIF2a and CHOP) and ER chaperone/foldases (Bip, Ero1L, and PDI) were dramatically reduced in the hypothalamus in high fat diet-induced obese mice (Figure 3A). Furthermore, mRNA expression levels of ER stress responsive genes (*Xbp-1s*, *Atf4*, *Chop*, *Grp78*, and *Erdj4*) were significantly reduced in ADE-administrated high fat diet-induced obese mice compared with vehicle control (Figure 3B). However, low-fat-diet-induced mice showed no changes due to lack of ER stress (Supplementary Figures S2A,B and S3). These results indicate that ADE can forcefully reduce ER stress induced by a high-fat diet in the hypothalamus.

### 3.4. Central Administration of ADE Regulates Appetite through MAPK and mTOR Signaling

**Figure 4:**
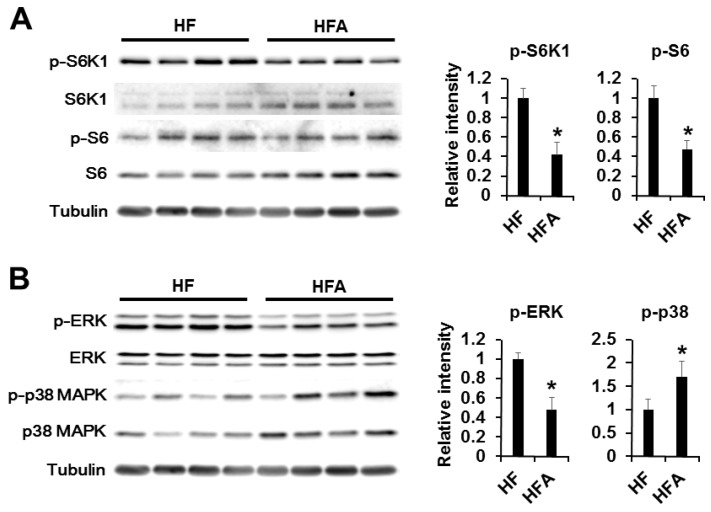
Central administration of ADE reduces ghrelin signaling through mTOR and ERK signaling pathways in mice fed a high-fat diet. (**A**) Effects of ICV administration of ADE (1 μL of 10 mg/mL) on mTOR signaling pathways. The results of densitometric analysis (right) are means ± SDs (*n* = 10 per group); * *p* values of < 0.05 indicate significant difference from administration with DMSO (1 μL of 20% DMSO); (**B**) Effects of ICV administration of ADE (1 μL of 10 mg/mL) on MAPK signaling pathways. The results of densitometric analysis (right) are means ± SDs (*n* = 10); * *p* values of < 0.05 indicate significant difference from administration with DMSO (1 μL of 20% DMSO). HF, high-fat diet with DMSO. HFA, high-fat diet with ADE.

Recent evidence has demonstrated that hypothalamic mammalian target of rapamycin (mTOR), a highly conserved serine-threonine kinase, signaling plays a role in modulation of feeding behavior by acting as a cellular sensor of changes in energy balance, nutrients, and growth factors [21,22]. Leptin and ghrelin seem to be powerful factors in the regulation of food intake and body weight. In the hypothalamus, activation of leptin or ghrelin receptor initiates different signaling cascades regulating food intake [23,24,25,26]. mTOR and MAPKs (ERK and p38) signaling has been previously associated with ghrelin [27,28,29,30,31]. For example, central administration of ghrelin has been reported to activate mTOR and ERK signaling to induce food intake. Therefore, we determined which signaling cascades are linked to appetite control after central administration of ADE. Firstly, we investigated leptin signaling with phospho-Stat3, -JAK2, and SOCS3 antibody. However, phosphorylation or expression of these proteins was unchanged. Secondly, we determined ghrelin signaling downstream of mTOR (phospho-S6K1 and -S6) and MAPKs (phospho-ERK and -p38) (Figure 4 and Supplementary Figure S4). As shown in Figure 4, S6K1, S6, and ERK phosphorylation levels were significantly reduced after administration of ADE compared with vehicle control. However, phosphorylation of p38MAPK was elevated after ADE administration in mice fed a high-fat diet. These results assume that ADE reduces appetite by antagonizing ghrelin signaling cascades rather than leptin signaling, and mTOR and MAPK signaling pathways were necessary for appetite regulation of ADE in the hypothalamus.

## 4. Discussion

The hypothalamus is considered a key player in the regulation of food intake and body weight, although hypothalamic dysfunction may occur in a chronic energy excess state [20,32]. Under obese conditions induced by pharmacologic or genetic causes, endoplasmic reticulum (ER) stress in the hypothalamus causes central leptin and insulin resistance, resulting in increased food intake, hypertension, and glucose intolerance, whereas reduction of ER stress significantly attenuates these metabolic derangements [33,34]. Beetle species, a very well-known insect, is widely used in Oriental medicine to treat various diseases such as diabetes and hepatofibrosis in Asian countries [8,35]. Insects can be used as a health food supplement or functional food, as they are rich in protein, vitamins, minerals, fiber, and unsaturated fatty acids [36,37,38]. Previous works reported that several kinds of insect extracts could be used as anti-obesity and liver disease treatment agents [7,9,39].

In this study, we demonstrated the functional effects of ADE on ER stress and appetite regulatory neuropeptide processing in obesity. In diet-induced obesity (DIO), the appetite regulatory α-melanocyte-stimulating hormone (α-MSH) is downregulated, whereas appetite-inducing neuropeptide Y (NPY) and aqouti-related protein (AgRP) are induced [18,19,20]. To study the effects of ADE on appetite control, ADE was injected with ICV, and food intake and body weight changes were measured (Figure 2A,B). As a result, food intake and body weight were markedly reduced after ADE administration compared with vehicle control, which was evident 2 h after infusion and was consistent after 24 h. Furthermore, we quantified the mRNA expression levels of *Npy*, *Agrp*, and *Pomc* by quantitative PCR. As shown in Figure 2C, *Npy* and *Agrp* mRNA expression levels were reduced, whereas *Pomc* mRNA level increased after ADE administration. From these results, we assume that the appetite reducing effect of ADE is mediated by restoration of DIO-induced leptin resistance accompanied by reduction of ER stress on the hypothalamus. Thus, we investigated expression levels of ER stress markers and chaperones using Western blot analysis or quantitative PCR. As shown in Figure 3, ER stress markers and chaperones were dramatically downregulated upon ADE infusion, whereas leptin resistance was not restored.

Ghrelin expression was elevated upon fasting and reduced upon feeding in normal and genetically obese rodents [40,41,42]. Based on these findings, we speculate that reduction of food intake and body weight by ADE may be due to antagonization of ghrelin function. In previous works, ICV injection of ghrelin induced orexigenic action by increasing mTOR and ERK signaling pathways [27,28,43,44]. Additionally, ghrelin was shown to inhibit p38 MAPK activation in oligodendrocytes and other types of cells [30,31]. Therefore, we determined levels of S6K1 and S6 phosphorylation, downstream signaling cascades of mTOR, and phosphorylation of MAPKs (ERK and p38) (Figure 4). Central administration of ADE significantly downregulated phosphorylation of S6K1, S6, and ERK as well as upregulation of p38 MAPK in mice fed a high-fat diet. However, low-fat-diet-induced mice showed no changes (Supplementary Figure S4). These result indicate that ADE reduces appetite by antagonizing ghrelin signaling cascades rather than leptin signaling, and mTOR and ERK signaling are necessary for appetite regulation of ADE in the hypothalamus in mice fed a high-fat diet.

In summary, our most significant finding is that ethanol extract of *Allomyrina dichotoma* larvae (ADE) has an anorexigenic effect through regulation of mTOR and MAPK pathways, resulting in reduced food intake and body weight changes accompanied by reduced ER stress. We speculate that the anorexigenic effect of ADE is due to antagonization of ghrelin-induced feeding behavior. Furthermore, ADE showed the strongest reducing effect on ER stress both *in vitro* and *in vivo* (Figure 3 and Supplementary Figure S2). Accumulating evidence suggests that chronic activation of ER stress contributes to the pathogenesis of many diseases, including neurodegenerative diseases, bipolar disorder, diabetes mellitus, atherosclerosis, inflammation, ischemia, heart diseases, liver and kidney disease, and cancer [45,46]. As research on ER stress-related diseases has recently increased, these results strongly suggest that ADE is available and valuable to use for treatment of various diseases driven by prolonged ER stress. This study demonstrates the anorexigenic and ER stress-reducing effects of ADE in the central nervous system and provides a strong possibility for the implications of insect-derived multiple compounds for the therapeutic purposes in patients. However, this study was limited to the administration of ADE, an anti-obesity drug, via the third ventricle, which grants further investigation to validate the effects via other routes of administration including oral. Furthermore, fractionation of ADE is also warranted to examine the proper components of the ADE responsible for these effects, as well as to provide strong evidence of the anorexigenic and ER stress-reducing effects of ADE on hypothalamic ER stress-driven metabolic disorders.

## 5. Conclusions

Intracerebroventricular (ICV) administration of the ADE had an antagonizing effect on ghrelin-induced feeding behavior through mTOR and MAPK signaling pathways. These findings strongly suggest that ADE and its constituent bioactive compounds are available and valuable to use for treatment of various diseases driven by prolonged ER stress

## Figures and Tables

**Table 1:** DNA primers for PCR.

Mouse cDNAs	Primer Sequences	GenBank Accession No.
**For Real-time PCR**		
*Xbp-1s*	Forward, 5′-AGGCTTGGTGTATACATGG-3′ Reverse, 5′-GGTCTGCTGAGTCCGCAGCAGG-3′	NM_013842
*Atf4*	Forward, 5′-GCAAGGAGGATGCCTTTTC-3′ Reverse, 5′-GTTTCCAGGTCATCCATTCG-3′	NM_009716
*Chop*	Forward, 5′-CCACCACACCTGAAAGCAGAA-3′ Reverse, 5′-AGGTGAAAGGCAGGGACTCA-3′	NM_007837
*Grp78*	Forward, 5′-GGCCTGCTCCGAGTCTGCTTC-3′ Reverse, 5′-CCGTGCCCACATCCTCCTTCT-3′	NM_022310
*Erdj4*	Forward, 5′-CCCCAGTGTCAAACTGTACCAG-3′ Reverse, 5′-AGCGTTTCCAATTTTCCATAAATT-3′	NM_013760
*Agrp*	Forward, 5′-TAGATCCACAGAACCGCGAGT-3′ Reverse, 5′-GAAGCGGCAGTAGCACGTA-3′	NM_007427
*Npy*	Forward, 5′-CTCCGCTCTGCGACACTAC-3′ Reverse, 5′-AGGGTCTTCAAGCCTTGTTCT-3′	NM_023456
*Pomc*	Forward, 5′-CTGGAGACGCCCGTGTTTC-3′ Reverse, 5′-TGGACTCGGCTCTGGACTG-3′	NM_001278584
*Socs3*	Forward, 5′-GAGTACCCCCAAGAGAGCTTACTA-3′ Reverse, 5′-CTCCTTAAAGTGGAGCATCATACTG-3′	NM_007707
*Gapdh*	Forward, 5′-CTTCAACAGCAACTCCCACTCTTCC-3′ Reverse, 5′-TGGGTGGTCCAGGGTTTCTTACTCCTT-3′	NM_001289726
**For Traditional PCR**		
*Xbp-1*	Forward, 5′-CAACCAGGAGTTAAGAACACG-3′ Reverse, 5′-AGGCAACAGTGTCAGAGTCC-3′	NM_013842
*Atf4*	Forward, 5′-GACCTGGAAACCATGCCAGA-3′ Reverse, 5′-TGGCTGCTGTCTTGTTTTGC-3′	NM_009716
*Chop*	Forward, 5′-TCCCCAGGAAACGAAGAGGA-3′ Reverse, 5′-TTGAGCCGCTCGTTCTCTTC-3′	NM_007837
*Gapdh*	Forward, 5′-GACCACAGTCCATGCCATCA-3′ Reverse, 5′-CATTGAGAGCAATGCCAGCC-3′	NM_001289726

## Supplementary Materials

The following are available online at http://www.mdpi.com/2072-6643/8/2/100/s1: Figure S1: Effects of central administration of ADE on food intake and body, Figure S2: Effects of ADE on ER stress responsive marker expression, Figure S3: Effects of central administration of ADE on ER stress responsive markers and ER chaperone/foldases expression in mice fed a low-fat diet, Figure S4: Central administration of ADE reduces ghrelin signaling through mTOR and ERK signaling pathways in mice fed a low-fat diet.
