# Supplementary material for: Allomyrina Dichotoma Larvae Regulate Food Intake and Body Weight in High Fat Diet-Induced Obese Mice Through mTOR and Mapk Signaling Pathways
Source: Nutrients. 2016 Feb 18;8(2):100. doi: 10.3390/nu8020100 (PMC4772062; doi:10.3390/nu8020100)
Supplement: Supplementary file 1 [file nutrients-08-00100-s001.ppt]

## Slide 1
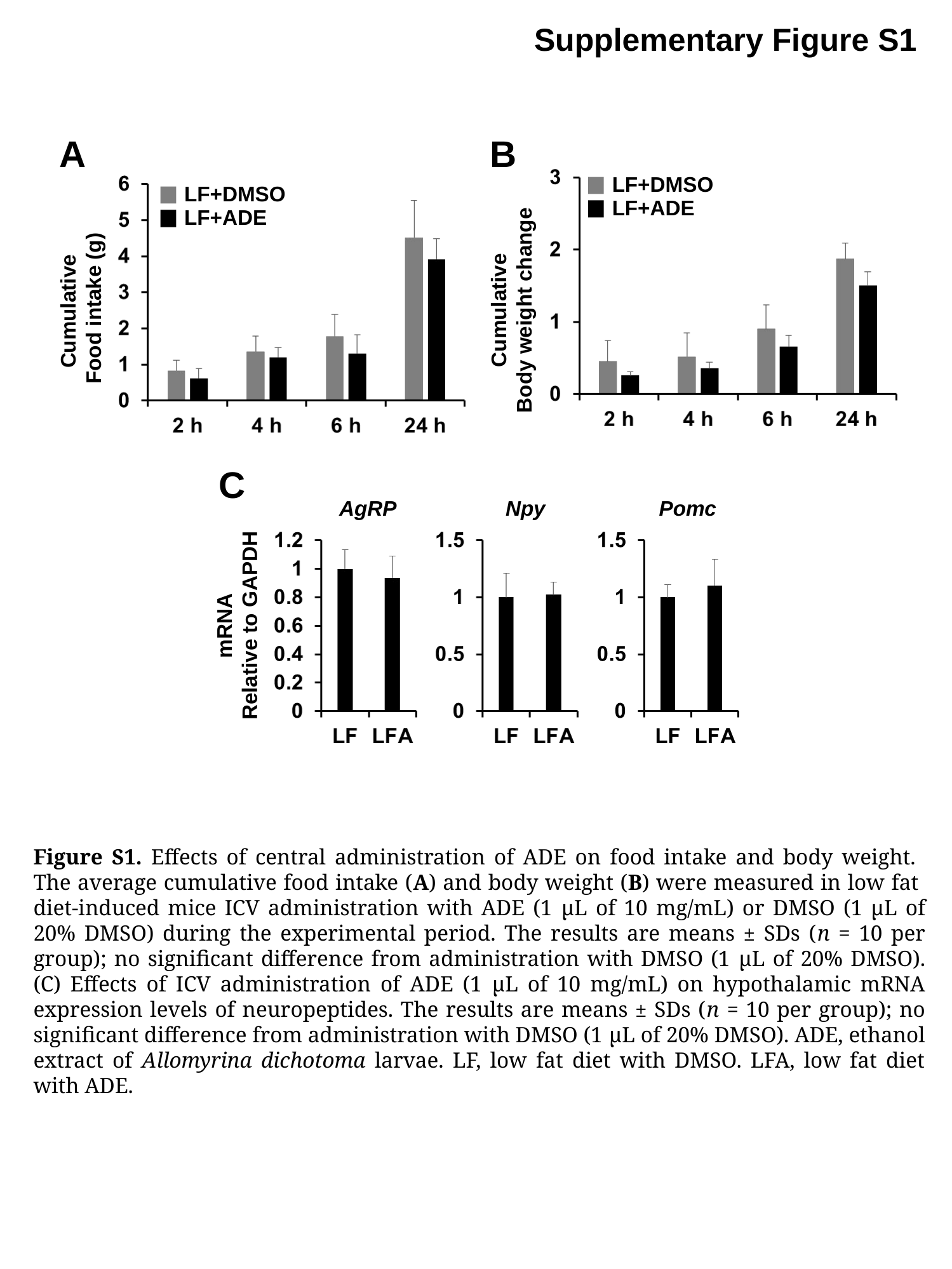

Supplementary Figure S1
A
B
LF+DMSO
LF+DMSO
LF+ADE
LF+ADE
Cumulative
Food intake (g)
Cumulative
Body weight change
C
AgRP
Npy
Pomc
mRNA
Relative to GAPDH
Figure S1. Effects of central administration of ADE on food intake and body weight.
The average cumulative food intake (A) and body weight (B) were measured in low fat
diet-induced mice ICV administration with ADE (1 μL of 10 mg/mL) or DMSO (1 μL of 20% DMSO) during the experimental period. The results are means ± SDs (n = 10 per group); no significant difference from administration with DMSO (1 μL of 20% DMSO). (C) Effects of ICV administration of ADE (1 μL of 10 mg/mL) on hypothalamic mRNA expression levels of neuropeptides. The results are means ± SDs (n = 10 per group); no significant difference from administration with DMSO (1 μL of 20% DMSO). ADE, ethanol extract of Allomyrina dichotoma larvae. LF, low fat diet with DMSO. LFA, low fat diet with ADE.

## Slide 2
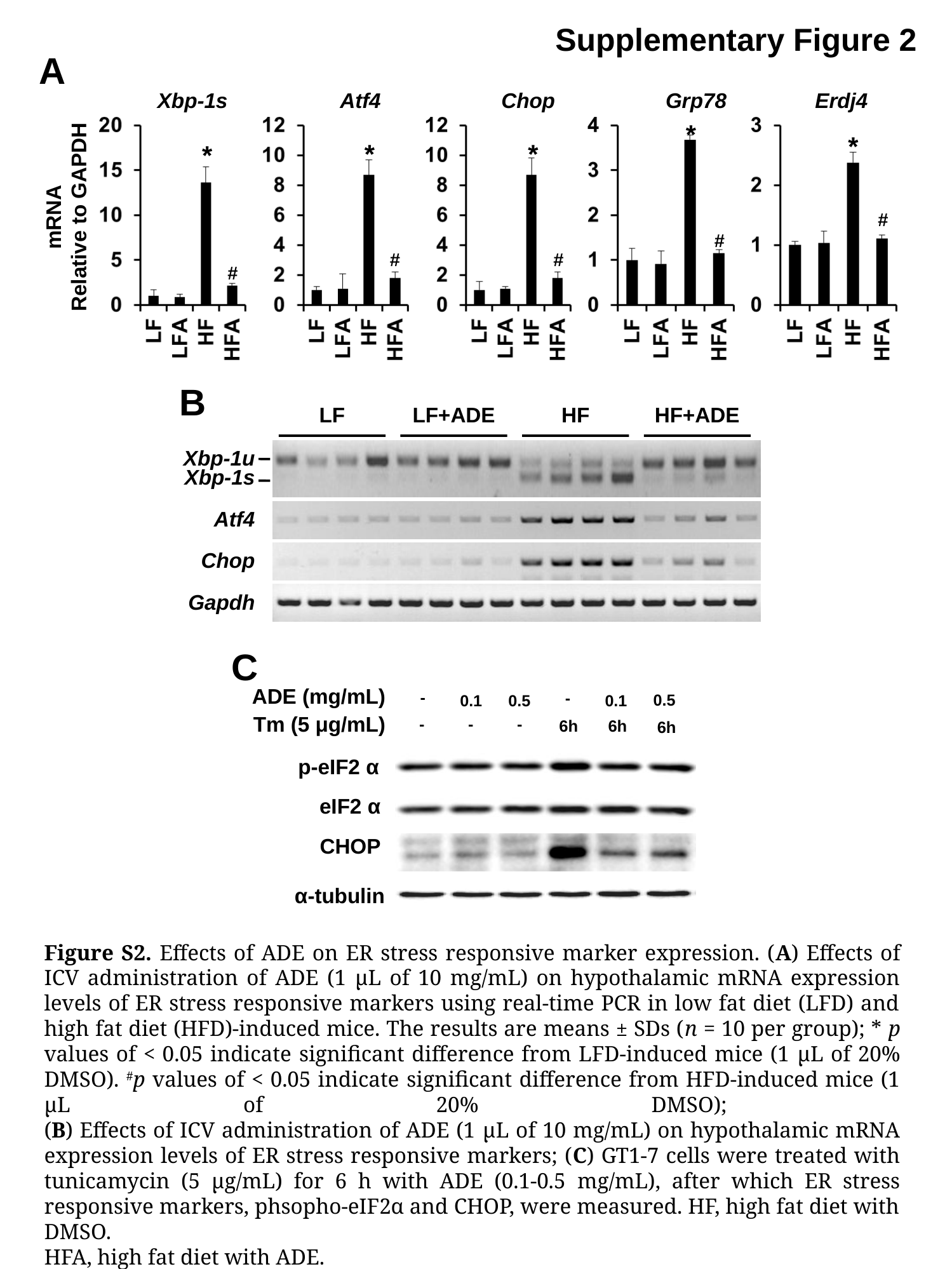

Supplementary Figure 2
A
Xbp-1s
Atf4
Chop
Grp78
Erdj4
*
*
*
*
*
mRNA
Relative to GAPDH
#
#
#
#
#
B
LF
LF+ADE
HF
HF+ADE
Xbp-1u
Xbp-1s
Atf4
Chop
Gapdh
C
ADE (mg/mL)
-
-
0.5
0.1
0.5
0.1
 Tm (5 μg/mL)
-
-
-
6h
6h
6h
p-eIF2 α
eIF2 α
CHOP
α-tubulin
Figure S2. Effects of ADE on ER stress responsive marker expression. (A) Effects of ICV administration of ADE (1 μL of 10 mg/mL) on hypothalamic mRNA expression levels of ER stress responsive markers using real-time PCR in low fat diet (LFD) and high fat diet (HFD)-induced mice. The results are means ± SDs (n = 10 per group); * p values of < 0.05 indicate significant difference from LFD-induced mice (1 μL of 20% DMSO). #p values of < 0.05 indicate significant difference from HFD-induced mice (1 μL of 20% DMSO);
(B) Effects of ICV administration of ADE (1 μL of 10 mg/mL) on hypothalamic mRNA expression levels of ER stress responsive markers; (C) GT1-7 cells were treated with tunicamycin (5 μg/mL) for 6 h with ADE (0.1-0.5 mg/mL), after which ER stress responsive markers, phsopho-eIF2α and CHOP, were measured. HF, high fat diet with DMSO.
HFA, high fat diet with ADE.

## Slide 3
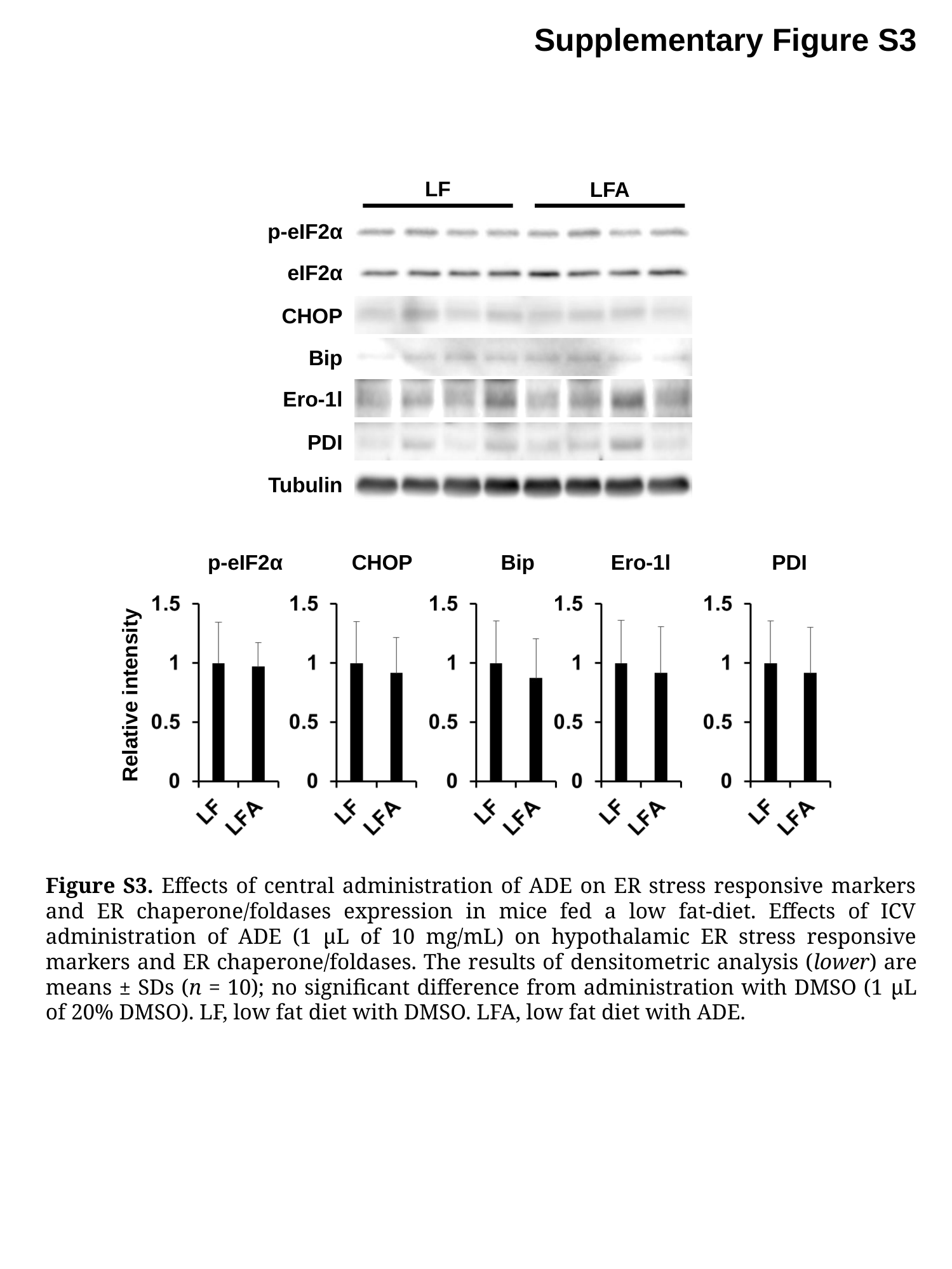

Supplementary Figure S3
LF
LFA
p-eIF2α
eIF2α
CHOP
Bip
Ero-1l
PDI
Tubulin
p-eIF2α
CHOP
Bip
Ero-1l
PDI
Relative intensity
Figure S3. Effects of central administration of ADE on ER stress responsive markers and ER chaperone/foldases expression in mice fed a low fat-diet. Effects of ICV administration of ADE (1 μL of 10 mg/mL) on hypothalamic ER stress responsive markers and ER chaperone/foldases. The results of densitometric analysis (lower) are means ± SDs (n = 10); no significant difference from administration with DMSO (1 μL of 20% DMSO). LF, low fat diet with DMSO. LFA, low fat diet with ADE.

## Slide 4
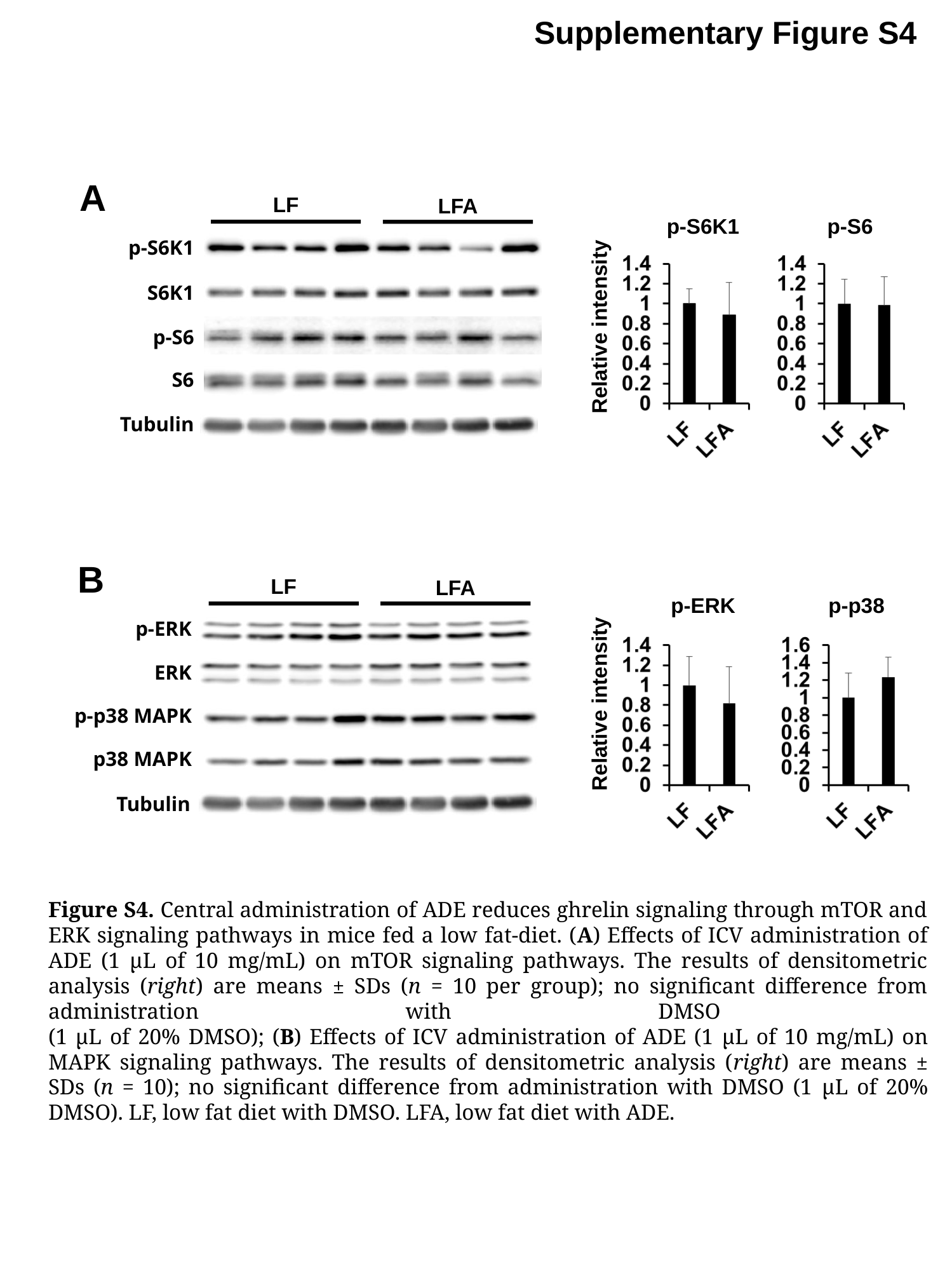

Supplementary Figure S4
A
LF
LFA
p-S6K1
p-S6
p-S6K1
S6K1
Relative intensity
p-S6
S6
Tubulin
B
LF
LFA
p-ERK
p-p38
p-ERK
 ERK
Relative intensity
p-p38 MAPK
p38 MAPK
Tubulin
Figure S4. Central administration of ADE reduces ghrelin signaling through mTOR and ERK signaling pathways in mice fed a low fat-diet. (A) Effects of ICV administration of ADE (1 μL of 10 mg/mL) on mTOR signaling pathways. The results of densitometric analysis (right) are means ± SDs (n = 10 per group); no significant difference from administration with DMSO
(1 μL of 20% DMSO); (B) Effects of ICV administration of ADE (1 μL of 10 mg/mL) on MAPK signaling pathways. The results of densitometric analysis (right) are means ± SDs (n = 10); no significant difference from administration with DMSO (1 μL of 20% DMSO). LF, low fat diet with DMSO. LFA, low fat diet with ADE.
